# An Open Pilot Study of the Effect and Tolerability of Add-On Multivitamin Therapy in Patients with Intractable Focal Epilepsy

**DOI:** 10.3390/nu12082359

**Published:** 2020-08-07

**Authors:** Hui Hua Chang, Pi-Shan Sung, Wei Chen Liao, Alice Y. W. Chang, Ya-Hsin Hsiao, Tzu-Fun Fu, Chin-Ying Huang, Chin-Wei Huang

**Affiliations:** 1 Institute of Clinical Pharmacy and Pharmaceutical Sciences, College of Medicine, National Cheng Kung University, Tainan 701, Taiwan; huihua@mail.ncku.edu.tw (H.H.C.); weizhen60@gmail.com (W.C.L.); 2 School of Pharmacy, College of Medicine, National Cheng Kung University, Tainan 701, Taiwan; 3 Department of Pharmacy, National Cheng Kung University Hospital, College of Medicine, National Cheng Kung University, Tainan 701, Taiwan; 4 Department of Pharmacy, National Cheng Kung University Hospital, Dou-Liou Branch, Yunlin 640, Taiwan; 5 Department of Neurology, National Cheng Kung University Hospital, College of Medicine, National Cheng Kung University, Tainan 701, Taiwan; pishansung@gmail.com; 6 Department of Physiology, College of Medicine, National Cheng Kung University, Tainan 701, Taiwan; aywchang@mail.ncku.edu.tw; 7 Institute of Basic Medical Sciences, College of Medicine, National Cheng Kung University, Tainan 701, Taiwan; yahsin@mail.ncku.edu.tw (Y.-H.H.); tffu@mail.ncku.edu.tw (T.-F.F.); 8 Institute of Behavioral Medicine, College of Medicine, National Cheng Kung University, Tainan 701, Taiwan; 9 Department of Pharmacology, College of Medicine, National Cheng Kung University, Tainan 701, Taiwan; 10 Department of Medical Laboratory Science and Biotechnology, College of Medicine, National Cheng Kung University, Tainan 701, Taiwan; 11 Department of Nutritional Services, National Cheng Kung University Hospital, Tainan 704, Taiwan; jagamitra.h@gmail.com

**Keywords:** multivitamin, antiepileptic drug, intractable epilepsy, effect, tolerability

## Abstract

Observational studies have investigated the potential modulatory effect of neuronal excitability by vitamins in epilepsy. We aimed to investigate whether the addition of multivitamin therapy (B6/B9, D, E and Q) to regular antiepileptic drug therapy could ameliorate seizures in patients with refractory focal epilepsy. We conducted a prospective cohort open study to investigate the effect and tolerability of add-on multivitamin therapy (daily dose: B6 100 mg, B9 5 mg, D 1000 IU, E 400 IU and coenzyme Q10 100 mg) in patients with intractable focal epilepsy. All patients had effect and safety assessments at baseline and after one, three and six months of the supplementation. Thirty patients (11 men and 19 women) with a mean age of 42.37 ± 9.40 years were recruited and four patients discontinued. The seizure frequency significantly decreased after the six-month supplementation (9.04 ± 18.16/month and 2.06 ± 3.89/month, *p* = 0.045). At the final visit, 62.5% of the patients showed a ≥50% reduction in seizure frequency, and 12.5% were seizure-free. As to safety and tolerability, most patients did not experience significant adverse events, although three patients reported seizure worsening. In conclusion, this pilot study demonstrated the therapeutic potential and essentially good tolerability of add-on multivitamin therapy in patients with refractory focal epilepsy.

## 1. Introduction

Epilepsy is a chronic neurological disorder that affects approximately 1% of the population globally. Despite advances in antiepileptic drugs (AEDs) and patient-tailored surgery, around 30% of patients still have uncontrolled epilepsy [1]. Many patients who achieve seizure control with AEDs suffer from serious adverse effects including sedation and cognitive impairment [2]. Therefore, despite the beneficial effects of the currently available AEDs, there is still a need for alternative therapy with different mechanisms and fewer adverse effects.

Previous studies have investigated the association between nutrients and seizures, and reported that micronutrient deficiencies may have a causal role in epilepsy. However, few observational studies have investigated whether metabolism and modulation of neuronal excitability by vitamins, especially B, D, E and Q, could potentially be effective in treating neuronal hyperexcitability disorders such as epilepsy and even epileptogenesis. Pyridoxal 5’-phosphate (PLP) is essential for the metabolism and synthesis of amino acids, gluconeogenesis, hematopoiesis, hormone regulation, immunologic functions and synthesis of neurotransmitters, including γ-aminobutyric acid (GABA). PLP deficiency may result in the lowering of seizure thresholds by impairing the synthesis of GABA [3]. In support of this hypothesis, Dave et al. reported selective pyridoxine deficiency in 94% of patients with status epilepticus [4].

Folic acid, vitamin B9, plays an important role in cell division and amino and nucleic acid synthesis. It is necessary for the normal development of the fetal spine, brain and skull. Ali et al. demonstrated that the addition of folic acid to AED therapy may have additional benefits on seizure control and memory [5], and Rasic-Markovic et al. reported that subchronic supplementation with folic acid and L-arginine had an antiepileptic effect in homocysteine thiolactone-induced epilepsy [6]. Vitamin D has been reported to exert anti-inflammatory actions, increase seizure threshold, and upregulate glutathione, which scavenges oxidative byproducts and chelates heavy metals [7,8]. Cholecalciferol has been found to significantly raise the electroconvulsive threshold and potentiate the anticonvulsant activity of some AEDs [9]. In addition, Hollo et al. reported a significant reduction in median seizure frequency by up to 40% in patients taking vitamin D3 supplementation, and they concluded that the normalization of serum vitamin 25(OH)D level has an anticonvulsant effect [7].

Vitamin E, a potent antioxidant, has been demonstrated to alter neuronal excitability. A double-blind and placebo-controlled study using 400 IU of vitamin E found a dramatic decrease in seizure activity in patients taking vitamin E supplements for three months [10]. Vitamin E administration has also been shown to cause a significant decrease in the frequency of seizures and improve electroencephalography (EEG) findings [11]. Vitamin Q (coenzyme Q10; CoQ10) is an antioxidant compound that exhibits a wide range of therapeutic effects that are attributed to its potent antioxidant property. The subchronic administration of CoQ10 has been reported to attenuate seizures induced by pentylenetetrazole or electroshock seizure models [12,13]. CoQ10 has also been shown to be a safe and effective adjuvant to phenytoin therapy for patients with epilepsy, both to ameliorate seizure severity and to protect against seizure-induced oxidative damage [14].

Vitamins are relatively safe in terms of adverse effects compared to standard AEDs. Although some evidence has shown that individual vitamins may be beneficial for epilepsy, clinical trials regarding multivitamins in patients with intractable epilepsy are lacking. Therefore, this study aimed to investigate whether the addition of multivitamin therapy (B6/B9, D, E and Q) to regular AED therapy could further ameliorate seizures in patients with refractory focal epilepsy, and to assess the long-term safety and tolerability of multivitamin therapy.

## 2. Materials and Methods

This was a prospective cohort study on the effect and tolerability of add-on vitamin therapy in patients with refractory epilepsy receiving regular treatment with AEDs. All subjects gave their informed consent for inclusion before they participated in the study. The study was conducted in accordance with the Declaration of Helsinki, and the protocol was approved by the ethics committee of the Institutional Review Board of National Cheng Kung University Hospital (B-BR-105-098), Tainan, Taiwan. Drug-resistant epilepsy was defined as failure of adequate trials of two tolerated, appropriately chosen and used AEDs (either as monotherapy or in combination) to achieve sustained seizure freedom [15].

### 2.1. Patients

The inclusion criteria were patients aged 18 years or older with a diagnosis of refractory focal epilepsy (according to the International League Against Epilepsy (ILAE) classification) who fulfilled all the following: drug-resistant epilepsy as defined above [15]; receiving concomitant AED(s) without dose adjustment within 4 weeks prior to the baseline period; having one or more seizures during the 4-week baseline period; no progressive or expansive brain injuries as previously documented by computed tomography (CT), magnetic resonance imaging (MRI) or other applicable imaging test in the past 3 years; and being willing and able to provide written informed consent. Patients were excluded from the study for any of the following reasons: history of status epilepticus within three months prior to the baseline period; history of active central nervous system infections, pseudoseizures, conversion disorders or other non-epileptic ictal events which could be confused with seizures within the past year; any conditions or diseases that an investigator considered would render a patient unsuitable to participate in the study; use of any central nervous system medication(s) requiring frequent dose adjustments with the exception of those on a stable dose for at least 4 weeks; history of alcoholism, drug abuse or drug addiction within the past year; and females who were pregnant/lactating or planning to become pregnant. For the patients who were already taking vitamins, we asked them to discontinue taking them for at least 1 month before entry into this study and during the study period, to avoid taking other vitamins, and to only take the vitamins provided by us to ensure a more precise evaluation and avoid toxicity.

After the patients had signed informed consent, baseline data on demographics, medical history, previous antiepileptic therapy, adjunct medication and diseases were collected. The patients’ seizure status during the three months prior to the baseline visit was recorded based on patient diaries, written patient records or detailed interviews.

### 2.2. Treatment, Evaluation and Follow up

The eligible patients were given the following multivitamins (with the daily dosage): B6 100 mg, folic acid (B9) 5 mg, D 1000 IU, E 400 IU and CoQ10 100 mg/day. The effectiveness of add-on vitamin treatment was assessed at one, three and six months based on patient diaries. Serum vitamin levels were determined at baseline, one, three and six months. EEG was performed at baseline and after three months. At these visits, the physicians also documented dosing, adverse events, retention and treatment satisfaction. Adverse effects were carefully monitored systemically, and if the patients encountered serious side effects such as allergies deemed to be associated with vitamins, the vitamin treatment was discontinued immediately.

All concomitant medications other than AEDs, and significant non-drug therapies such as surgery, blood transfusions and physical therapy, administered after the subjects entered in this study were documented. The patients who used traditional AEDs (phenytoin, carbamazepine, valproic acid, clonazepam) had their therapeutic drug level monitored at baseline and after one, three and six months. The baseline AEDs were not changed during the six months of trial period.

### 2.3. Outcome Criteria

The primary outcome criterion was the decrease in seizure frequency. Patients were considered to be “responders” if they experienced a decrease in seizure frequency by at least 50% during the treatment period. Seizure frequency and severity data were collected using seizure diaries kept by the patients themselves. The secondary outcome measure was reported treatment-emergent adverse effects.

### 2.4. Serum Vitamin Levels

Serum vitamin levels were determined at baseline and after one, three and six months of multivitamin supplementation. The concentrations of the vitamins were examined using an electrochemiluminescence immunoassay (ECLIA) or an enzyme-linked immunosorbent assay (ELISA) following the manufacturer’s instructions, including vitamin B6 (MyBioSource, Inc., San Diego, CA, USA), vitamin B9 (cobas^®^, Roche Diagnostics GmbH, Mannheim, Germany), vitamin D (cobas^®^, Roche Diagnostics GmbH, Mannheim, Germany), vitamin E (MyBioSource, Inc., San Diego, CA, USA) and CoQ10 (Cusabio^®^ Biotech Co., Ltd., Wuhan, China).

### 2.5. Metabolic Profile

We also evaluated the patients’ metabolic profiles at baseline and after six months of taking the supplementations, including: body profile: body weight (BW, kg), body mass index (kg/m^2^), waist circumference (cm); lipid profile: cholesterol (mg/dL), high-density lipoprotein (mg/dL), low-density lipoprotein (mg/dL) and triglycerides (mg/dL); and glucose profile: AC glucose (mg/dL), insulin (uIU/mL), Homeostatic Model Assessment of Insulin Resistance (HOMA-IR), McAuley Index and HbA1c (%).

The McAuley Index was used to predict insulin resistance in normoglycemic individuals [16]. It has been shown to have high sensitivity and specificity [17], and to be suitable for epidemiological/research purposes [16].

$$\mathrm{McAuley}\;\mathrm{Index}=e^{\left[2.63-0.28\ln\;\mathrm{insulin}\;\mathrm{value}\;\left(\frac{\mathrm{mIU}}{L}\right)-0.31\;\ln\mathrm{triglycerides}\;\mathrm{value}\left(\frac{\mathrm{mmol}}{L}\right)\right]}$$

### 2.6. Compliance

The eight-item Morisky Medication Adherence Scale (MMAS-8) was used to collect information on the patients’ medication compliance [18]. Containing eight questions, this scale is used to evaluate the status and habits of taking medications during a test period [19]. In this study, we used the modified Taiwanese version which has been linguistically validated by experts. Besides, all patients enrolled in the study were asked to record the vitamins taken daily and return the record at the next visit.

### 2.7. Statistical Analysis

We analyzed data using the Statistical Package for Social Sciences version 25.0 (SPSS Inc., Chicago, IL, USA). All demographic and clinical characteristics of the patients were reported as either number and percentage for categorical variables, or mean ± standard deviation for continuous variables. The Student’s t-test and chi-square (χ^2^) test were used to evaluate differences in characteristics between groups. Associations were tested using Spearman’s correlation analysis, due to the results of normality tests for the variables. Generalized estimating equation analysis was used to estimate the trend of seizure frequency and treatment response rate after six months of multivitamin supplementation. All tests were two-sided, and a *p*-value <0.05 was considered to be statistically significant unless otherwise specified.

## 3. Results

Thirty patients were enrolled (11 men and 19 women). Four patients discontinued the study (three reported seizure worsening and took vitamins less than ten days, one decided to prepare for pregnancy) and one patient dropped at the third visit (included in the analysis). Finally, 26 patients (nine men and 17 women; age range 21–64 years; mean age 42.37 ± 9.40 years) were included in the subsequent analysis. The demographics are listed in Table 1. The mean age at onset of epilepsy was 29.92 ± 9.32 years. Regarding the type of seizure, most of the patients had both focal impaired awareness seizures (FIASs) and focal aware seizures (FASs) (46.15%), followed by FIASs and focal to bilateral tonic-clonic seizures (FBTCSs) (30.77%) and FIASs only (23.07%). Fifteen (57.70%) of the patients had non-lesional epilepsy and 15 (57.70%) had temporal lobe epilepsy. Fifteen patients (57.70%) had either right or left hemisphere focus, and six patients (23.08%) had unknown focus. Eighteen patients (69.23%) used more than three AEDs (Table 1). Levetiracetam was the most commonly used AED (14 patients, 53.84%).

### 3.1. Effectiveness on Seizure Frequency (Primary Outcome) and Serum Levels of Vitamins

**Figure 1:**
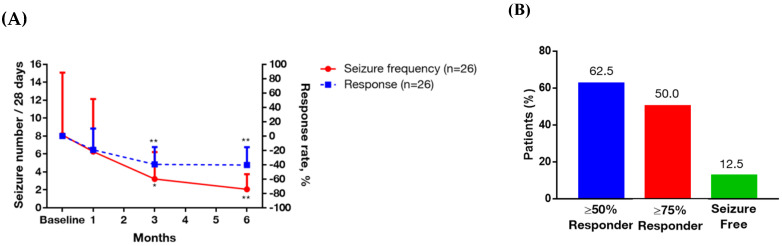
Trend of seizure frequency, treatment response rate and responder rate after six months of multivitamin supplementation in the patients with intractable focal epilepsy. Data are presented as mean ± 95% confidence interval (above). (**A**) Left y-axis represents seizure frequency, right y-axis represents treatment response rate, and x-axis displays time (by month). Significance was calculated using generalized estimating equation analysis, comparing baseline and after multivitamin supplementations. * *p* < 0.05, ** *p* < 0.01. (**B**) Responder rate. Percentage calculations are based on the number of subjects at the final visit.

The addition of multivitamins to the baseline AED therapy reduced the seizure frequency (9.04 ± 18.16 at baseline vs. 2.06 ± 3.89 after six months of supplementation, *p* < 0.05) (Table 2). The trend of seizure frequency and treatment response after six months of multivitamin supplementation in these patients are shown in Figure 1A, and demonstrated a significantly better response after three and six months compared to baseline. At the final visit, 62.5% of the patients showed a ≥50% reduction in seizure frequency from baseline, 50% showed a ≥75% reduction, and 12.5% were seizure-free (Figure 1B). The therapeutic drug monitoring of the patients who used traditional AEDs (phenytoin, carbamazepine, valproic acid, clonazepam) showed stable levels.

**Figure 2:**
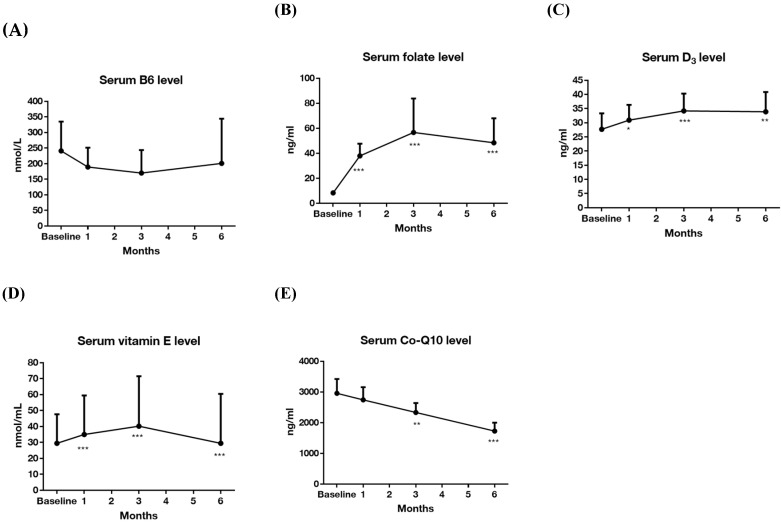
Changes in vitamin levels in the 6-month trial (*n* = 26). (**A**) Serum level of vitamin B6; (**B**) serum level of folate; (**C**) serum level of vitamin D3; (**D**) serum level of vitamin E; (**E**) serum level of coenzyme Q10. Data are presented as mean ± 95% confidence interval (above). Y-axis represents vitamin serum levels, and x-axis displays time (by month). Significance was calculated using generalized estimating equation analysis, comparing baseline and after vitamin supplementations. * *p* < 0.05, ** *p* < 0.01, *** *p* < 0.001.

Serum levels of vitamins are listed in Table 2. The baseline vs. after six-month supplementation for these vitamins were: B6 (nmol/L): 207.09 ± 215.64 vs. 200.75 ± 237.34 (*p* = 0.911); B9 (ng/mL): 8.39 ± 4.12 vs. 48.40 ± 32.38 (*p* = 0.001); D (ng/mL): 26.21 ± 13.64 vs. 33.90 ± 11.55 (*p* = 0.01); E (nmol/mL): 38.16 ± 51.19 vs. 29.47 ± 51.44 (*p* = 0.068); CoQ10 (ng/mL): 2947.61 ± 856.08 vs. 1725.32 ± 450.91 (*p* < 0.001). The serum levels of all five vitamins at baseline and after three and six months are presented in Figure 2. The B9 (folate) and E level during follow up were significantly higher than at baseline, and the CoQ10 levels at three and six months were significantly lower than at baseline. For those who were defined as responder, the vitamin level at baseline and after six months are shown in Table 3. The levels of folate, vitamin E and Q10 were significantly different between baseline and after six-month intervention (B9 (ng/mL): 8.58 ± 4.23 vs. 32.93 ± 9.27 (*p* < 0.001); E (nmol/mL): 30.24 ± 42.73 vs. 17.64 ± 29.07 (*p* = 0.028); CoQ10 (ng/mL): 2812.49 ± 842.07 vs. 1885.57 ± 514.14 (*p* < 0.001)).

Table 4 and Table 5 show the treatment outcomes of multivitamin supplementation in the patients. In the generalized estimating equations model, seizure frequency was significantly decreased after receiving multivitamin supplementation for over three months compared to baseline (*p* = 0.014 for three months and *p* = 0.003 for six months) (Table 4). Even in the model adjusted for compliance (Table 5), the results were consistent (*p* = 0.009 for both three months and six months). Moreover, the patients had a significantly better response rate after three months; the estimate (SE) for three months and six months were −39.36 ± 17.61 (*p* = 0.025) and −50.78 ± 17.48 (*p* = 0.004), respectively. The correlation of MMAS score and vitamin usage condition were presented in Table 6, and showed strong correlation between each other (*p* < 0.001). Table S1 shows the seizure frequency, treatment response and serum vitamin levels at baseline and one, three and six months. The correlations between serum vitamin levels are shown in Figure S1. Those who had a good treatment response had a higher vitamin D concentration.

### 3.2. Body and Metabolic Profiles

There were no significant differences in body weight, body mass index and waist circumference between baseline and after six months of multivitamin supplementation (Table 2). There was also no significant difference in lipid profile between baseline and after six months of multivitamin supplementation (Table 2). In terms of blood glucose profile, there were significant differences in HOMA-IR (1.98 ± 1.37 vs. 2.89 ± 2.40, *p* < 0.05), McAuley Index (8.31 ± 2.18 vs. 7.27 ± 1.76, *p* < 0.05) and HbA1c (5.24 ± 0.43 vs. 5.08 ± 0.43, *p* < 0.05) between baseline and after six months of multivitamin supplementation. No significant differences in AC glucose and insulin level were observed (Table 2).

### 3.3. Safety and Tolerability (Secondary Outcome)

With regards to safety and tolerability, most of the patients did not experience any significant treatment-emergent adverse events. Four patients experienced minor events, including one with dizziness (comedications: perampanel, levetiracetam and oxcarbazepine), one with insomnia (comedications: carbamazepine, perampanel, vigabatrin and zonisamide) and two with minor skin rashes (comedications: valproate and lamotrigine; carbamazepine, perampanel, topiramate and valproate). All of these adverse events resolved by two weeks. The three patients who reported worsening of seizures were all taking levetiracetam (one with concurrent perampanel, one with lamotrigine and one with levetiracetam monotherapy) (Table 7). The patient with levetiracetam monotherapy had an improvement in FIASs, although he had two FBTCSs after taking the multivitamin supplementation. The other two patients reported an increase in FIAS frequency (from once per month to two to three seizures per month) after taking the multivitamin supplementation.

Most of the patients who reported having adverse events were using sodium channel blockers concurrently, including carbamazepine, oxcarbazepine and lamotrigine. The two patients with skin rashes both used valproate, one with concurrent carbamazepine and the other one with lamotrigine.

## 4. Discussion

This pilot study on the treatment of patients with intractable epilepsy suggests that add-on multivitamin treatment can provide a clinically meaningful reduction in seizure frequency in the majority of patients with a tolerable adverse events profile. To the best of our knowledge, this is the first study on add-on multivitamin supplements to AED therapy in patients with intractable focal epilepsy. 

The pathomechanisms of intractable epilepsy are complicated, and probably involve different levels of genetic or symptomatic molecular and cellular alterations [20,21]. Despite advances in novel AEDs and patient-tailored surgery, around 30% of patients with intractable epilepsy remain uncontrolled [1,21]. Vitamins are pleiotropic and have anti-inflammatory and antioxidant effects, and the individual vitamins added on in this study have been shown to have antiseizure effects. In this study, the multivitamin supplementation clearly demonstrated treatment potential as an add-on to standard AED therapy. 

Three patients discontinued this study due to a subjective worsening of seizures within three months. These three patients all took levetiracetam. Whether there was any pharmacodynamic interaction between levetiracetam and the multivitamins remains to be determined, as most of the patients who did not experience worsening of seizures also took levetiracetam. Lovecchio et al. reported that the rapid intravenous administration of pyridoxine (5 g of pyridoxine (50 mL) over 5 min) might induce transient metabolic acidosis [22], however, this is quite different from our oral daily pyridoxine dosage (5 mg). Whether the worsening of seizures was related to the concentration changes in vitamins B9, D and E remains to be determined.

The serum levels of B9 and D after starting the supplementation for six months were significantly higher than their baseline levels. In contrast, the level of CoQ10 significantly decreased, and the serum level of vitamin E also tended to be reduced. This may be related to the cosupplementation of CoQ10 and vitamin E. A previous study in baboons reported that vitamin E tended to reduce plasma total CoQ10 concentrations, and that CoQ10 supplementation may also reduce plasma α-tocopherol (a type of vitamin E) concentration [23]. This may indicate that the cosupplementation of CoQ10 and vitamin E may have affected their plasma level. However, cosupplementation of CoQ10 has been reported to significantly enhance the anti-inflammatory effect of vitamin E irrespective of the reduction in detected plasma levels of CoQ10 and vitamin E [23]. 

Regarding the metabolic profile, our patients had a normal fasting glucose level both at baseline and after six months of supplementation. Although the insulin level had an increasing trend, HOMA-IR increased and McAuley Index decreased during the treatment, there was a decrease in HbA1c level. Our earlier studies on patients with diabetes and seizures suggested an association between glucose and seizure susceptibility, and correlations between the level of glucose, HbA1c and risk of seizure recurrence [24,25,26,27,28]. Although the patients in this study were not diabetic, the effectiveness in reducing seizures following add-on multivitamins was correlated with a reduction in HbA1c. Nevertheless, the increase in IR, as evidenced by the reduction in McAuley Index in our non-diabetic patients with epilepsy, suggests that further investigations are needed. Factors known to be associated with IR include cardiovascular disease, stress and carotid atherosclerosis [29,30,31]. In addition, an inverse relationship between serum folate level and markers of IR has been reported, which was not supported in our study on non-diabetic patients with epilepsy [32]. Another study also failed to establish an association between IR and homocysteine [33]. Studies on the effects of vitamin D on insulin sensitivity have been inconsistent [34,35]. Although it has been proposed that vitamin D deficiency plays an important role in IR [36], many factors are involved, including age, obesity and the presence of prediabetes as well as other cardiovascular risk factors [37]. Inconsistent results between vitamin E and IR have also been reported [38,39]. 

The plasma level of triglycerides tended to increase after six months of supplementation, although this trend was not significant in the other lipid profiles. The influence of multivitamins on lipid profiles may be complex, especially with regards to the cosupplementation of lipid-soluble vitamins. One randomized controlled trial showed that vitamin D may increase the level of total cholesterol, triglycerides, very low-density lipoprotein (VLDL), LDL and high-density lipoprotein, thus potentially having an unfavorable effect on lipid metabolism [40]. In contrast, supplementation of vitamin E and CoQ10 has been reported to possibly reduce low-density lipoprotein and triglycerides [41,42,43]. In the current study, the plasma level of vitamin D significantly increased, and was thus potentially related to the trend of increasing triglyceride level. However, the change in lipid profile was not significant, and mixed effects of the vitamins on lipid profiles may be possible. 

To facilitate good adherence to medications in our patients, especially when having to take several tablets at a time, we carefully explained the importance of compliance, and the results were evidenced by the vitamin blood levels and compliance scale. In addition, the patients taking traditional AEDs had stable serum therapeutic drug levels. Although the information regarding the effectiveness largely came from patient diaries and family reports, the information obtained from EEG and blood levels were generally supportive of the results of effectiveness, which were also consistent with the compliance review. Compliance may also be improved with a once-daily regimen in the future.

There are several limitations to this study, including the lack of a control group, open label design and lack of all AED concentrations. The lack of controls as a placebo group was due to the high technological and pharmaceutical requirement to develop a placebo capsule, as it needs to incorporate numerous vitamin tablets, including both water- and non-water-soluble vitamins, into one or two tablets, and furthermore, the vitamin dosages in this study are not typical dosages used on the market. To overcome this problem, we carefully reviewed the patients’ histories and primary outcome criteria of seizure frequency, using patient diaries, prior to starting multivitamin therapy. With regards to AED concentrations, although we could not obtain the newer AED concentrations, we did check the concentration of conventional AEDs (phenytoin, carbamazepine, valproic acid, clonazepam) and we did not see any clinically meaningful interactions between the vitamins and these conventional AEDs. Moreover, at each time point evaluation, we carefully reviewed seizure frequency, serum vitamin levels and EEG results to evaluate the consistency. Although this study was also limited by the relatively small sample size, we are confident that the historical cohort shed light on the effect and tolerability of add-on multivitamins in patients with intractable epilepsy. Further studies with control groups are important to confirm the results of this pilot study.

## 5. Conclusions

This pilot study demonstrated that add-on multivitamin supplementation to standard AED therapy had good association with seizure reduction and tolerability in patients with refractory focal epilepsy. Based on their relatively low cost and safety in terms of adverse effects, large-scale randomized clinical trials on multivitamins in patients with intractable epilepsy are warranted.

## Figures and Tables

**Table 1:** Baseline characteristics of the participants.

		Total (*n* = 26)
Female, *n* (%)	17	65.38%
Age (mean, SD), years	42.37	9.40
Age at onset of epilepsy (mean, SD), years	29.92	9.32
Type of seizure, *n* (%)	FIAS	6 (23.07%)
	FIAS and FAS	12 (46.15%)
	FIAS and FBTCS	8 (30.77%)
Negative MRI abnormality, *n* (%)		15 (57.70%)
Epilepsy syndrome, *n* (%)	Temporal lobe epilepsy	15 (57.70%)
	Extratemporal lobe epilepsy	11 (42.30%)
Side of EEG focus, *n* (%)	Left/right	10/5 (38.46%/19.23%)
	Bilateral	5 (19.23%)
	Unknown	6 (23.08%)
Patients with ≥3 AEDs,*n* (%)		18 (69.23%)
Concomitant AEDs, *n* (%)	Levetiracetam	14 (53.84%)
	Valproate	11 (42.31%)
	Perampanel	11 (42.31%)
	Clobazam	9 (34.62%)
	Topiramate	8 (30.77%)
	Lamotrigine	8 (30.77%)
	Clonazepam	5 (19.23%)
	Zonisamide	4 (15.38%)
	Carbamazepine	4 (15.38%)
	Vigabatrin	4 (15.38%)
	Phenytoin	2 (7.69%)
	Oxcarbazepine	1 (3.85%)

AED, antiepileptic drug; FIAS, focal impaired awareness seizure; FAS, focal awareness seizure; FBTCS, focal to bilateral tonic-clonic seizure; EEG, electroencephalography.

**Table 2:** Characteristics of the patients with intractable epilepsy at baseline and after 6 months of multivitamin supplementation (*n* = 26).

Characteristics	Normal Range (Adult)	Baseline	After 6 Months of Supplementation	Comparison		
		Mean ± SD	Mean ± SD	*t*/χ^2^	95% CI	*p*-Value
Seizure frequency, per 28 days		9.04 ± 18.16	2.06 ± 3.89	2.13	(0.18–13.78)	0.045 *
Serum vitamin level						
B6, nmol/L	20–202	207.09 ± 215.64	200.75 ± 237.34	0.11	(−115.09–−127.76)	0.911
B9, ng/mL	4.6–34.8	8.39 ± 4.12	48.40 ± 32.38	−4.31	(−60.24–−19.77)	0.001 **
D, ng/mL	30–100	26.21 ± 13.64	33.90 ± 11.55	−3.05	(−13.18–−2.20)	0.010 **
E, nmol/mL	11.6–39.5	38.16 ± 51.19	29.47 ± 51.44	2.01	(−0.73–18.13)	0.068
Q10, ng/mL	360–1590	2947.61 ± 856.08	1725.32 ± 450.91	5.98	(777.25–1667.33)	<0.001 ***
Body profile						
Body weight, kg		62.42 ± 14.17	63.20 ± 14.52	−1.45	(−1.89–0.33)	0.161
BMI, kg/m^2^		23.59 ± 4.51	23.92 ± 4.89	−1.65	(−0.76–0.09)	0.113
Waist circumference, cm		85.14 ± 8.16	85.34 ± 8.59	−0.23	(−2.08–1.67)	0.822
Lipid profile						
Cholesterol, mg/dL	<200	185.82 ± 28.79	180.91 ± 29.35	1.33	(−2.75–12.57)	0.197
HDL, mg/dL	>40	66.27 ± 17.59	67.18 ± 18.42	−0.49	(−4.78–2.96)	0.630
LDL, mg/dL	<100	115.82 ± 31.15	111.45 ± 32.61	1.77	(−0.76–9.48)	0.091
TG, mg/dL	<150	87.62 ± 43.97	104.05 ± 42.66	−1.94	(−34.08–1.22)	0.066
Sugar profile						
AC glucose, mg/dL	60–99	86.59 ± 7.03	90.09 ± 13.47	−1.72	(−7.73–0.73)	0.100
Insulin, uIU/mL	4–16	9.16 ± 6.15	12.50 ± 9.57	−2.05	(−6.73–0.05)	0.053
HOMA-IR	<1.9	1.98 ± 1.37	2.89 ± 2.40	−2.14	(−1.79–−0.03)	0.044 *
McAuley Index	<5.8	8.31 ± 2.18	7.27 ± 1.76	2.47	(0.16–1.93)	0.023 *
HbA1c, %	4.8–5.9	5.24 ± 0.43	5.08 ± 0.43	2.29	(0.01–0.30)	0.034 *

Data are presented as mean ± SD or number (percentage). AED, antiepileptic drug; B6, pyridoxal 5′-phosphate (PLP); B9, total folate; D, total 25 hydroxyvitamin D; E, total human vitamin E; Q10, total coenzyme Q10; BMI, body mass index; HDL, high-density lipoprotein cholesterol; LDL, low-density lipoprotein cholesterol; TG, triglyceride; AC glucose, glucose ante cibum; HOMA-IR, Homeostatic Model Assessment for Insulin Resistance; HbA1c, glycated hemoglobin A1c; * *p* < 0.05, ** *p* < 0.01, *** *p* < 0.001.

**Table 3:** The vitamin levels at baseline and after six-month supplementation in responders.

Characteristics	Baseline	After 6 Months of Supplementation	Comparison		
	Mean ± SD	Mean ± SD	*t*	95% CI	*p*-Value
Serum vitamin level					
B6, nmol/L	247.87 ± 228.00	163.93 ± 205.43	1.60	(−26.00–193.87)	0.126
B9, ng/mL	8.58 ± 4.23	32.93 ± 9.27	−10.20	(−29.36–−19.33)	<0.001 ***
D, ng/mL	26.53 ± 15.68	32.43 ± 11.05	−2.02	(−12.04–0.24)	0.059
E, nmol/mL	30.24 ± 42.73	17.64 ± 29.07	2.39	(1.51–23.69)	0.028 *
Q10, ng/mL	2812.49 ± 842.07	1885.57 ± 514.14	4.90	(529.38–1324.47)	<0.001 ***

Data are presented as mean ± SD or number (percentage). AED, antiepileptic drug; B6, pyridoxal 5′-phosphate (PLP); B9, total folate; D, total 25 hydroxyvitamin D; E, total human vitamin E; Q10, total coenzyme Q10. * *p* < 0.05, *** *p* < 0.001.

**Table 4:** Generalized estimating equations analysis of seizure frequency and treatment response rate after 6 months of multivitamin supplementation in the patients with intractable focal epilepsy (crude).

	Seizure Frequency (Per 28 Days) ^∀^ (*n* = 26)			Treatment Response Rate ^※^ (*n* = 26)		
Parameters	Estimate (SE)	Wald χ^2^	*p*-Value	Estimate (SE)	Wald χ^2^	*p*-Value
Intercept	8.12 ± 2.36	11.87	0.001 ***	0.00 ± 10.35	0.00	1.000
Visit						
6 months	−6.59 ± 2.21	8.88	0.003 **	−39.29 ± 13.07	9.04	0.003 **
3 months	−5.45 ± 2.21	6.06	0.014 *	−38.45 ± 13.07	8.66	0.003 **
1 month	−2.21 ± 2.18	1.03	0.310	−19.29 ± 12.89	2.24	0.135
Baseline (reference)	0			0		

Abbreviations: SE, standard error. * *p* < 0.05, ** *p* < 0.01, *** *p* < 0.001. **^∀^** Seizure frequency was defined as number of seizures within 28 days. **^※^** Treatment response rates were calculated as (seizure frequency after−seizure frequency baseline)/seizure frequency baseline.

**Table 5:** Generalized estimating equations analysis of seizure frequency and treatment response rate after 6 months of multivitamin supplementation in the patients with intractable focal epilepsy (adjusted compliance).

	Seizure Frequency (Per 28 Days) ^∀^ (*n* = 26)			Treatment Response Rate ^※^ (*n* = 26)		
Parameters	Estimate (SE)	Wald χ2	*p*-Value	Estimate (SE)	Wald χ^2^	*p*-Value
Intercept	10.99 ± 3.23	11.56	0.001 ***	5.08 ± 14.93	0.116	0.734
Visit						
6 months	−8.16 ± 3.14	6.76	0.009 **	−50.78 ± 17.48	8.44	0.004 **
3 months	−8.25 ± 3.15	6.86	0.009 **	−39.36 ± 17.61	5.00	0.025 **
1 month	−3.47 ± 3.03	1.31	0.253	−16.91 ± 17.02	0.99	0.320
Baseline (reference)	0			0		
Compliance ^#^	−3.66 ± 3.15	1.35	0.246	−8.12 ± 16.26	0.25	0.617
Visit Compliance						
6 months	1.68 ± 3.49	0.23	0.631	22.68 ± 19.35	1.37	0.241
3 months	3.99 ± 3.37	1.41	0.236	1.80 ± 18.72	0.01	0.923
1 month	0.75 ± 3.64	0.04	0.836	−18.39 ± 20.44	0.81	0.368
Baseline (reference)	0			0		

Abbreviations: SE, standard error. ** *p* < 0.01, *** *p* < 0.001. **^∀^** Seizure frequency was defined as number of seizures within 28 days. **^※^** Treatment response rates were calculated as (seizure frequency after−seizure frequency baseline)/seizure frequency baseline. ^#^ Compliance was determined by the total score of MMAS: (0) score = 8, high compliance; (1) score of 6 to <8, medium compliance; (2) score <6, low compliance.

**Table 6:** Correlation between MMAS scale and vitamin usage condition.

	MMAS (0~1 Month)	MMAS (1~3 Month)	MMAS (3~6 Month)
Vitamin usage (0~1 month)	−0.999 (*p* < 0.001 ***)	-	-
Vitamin usage (1~3 month)	-	−0.798 (*p* < 0.001 ***)	-
Vitamin usage (3~6 month)	-	-	−0.633 (*p* < 0.001 ***)

The correlation between MMAS score and vitamin usage record were calculated by Spearman’s correlation. Abbreviation: MMAS, Morisky Medication Adherence Scale. *** *p* < 0.001. The range of MMAS were scored 0 (low compliance) to 8 (high compliance). The vitamin usage condition was defined by the vitamin usage record: 0 = Took vitamins on time; 1 = Forgot to take vitamins ≤2 times per week; 2 = Forgot to take vitamins >2 times per week; 8 = Did not take vitamins for a period of time (gastroenteritis, go abroad, etc.); 9 = Discontinued because of seizure-related side effects.

**Table 7:** Adverse events reported in the patients, including the three patients who discontinued.

Adverse Event	Patients, *n* (%)	Comedications
Dizziness ^a^	1 (3.33)	PER, LEV, OXC
Insomnia ^a^	1 (3.33)	CBZ, PER, VGB, ZNS
Skin rashes ^a^	2 (6.67)	VPA, LTG
		CBZ, PER, TPM, VPA
Seizure worsening ^b^	3 (10)	LEV, PER
		LEV, LTG
		LEV

^a^ resolved by 2 weeks; ^b^ discontinued the study; PER, perampanel; LEV, levetiracetam; OXC, oxcarbazepine; CBZ, carbamazepine; VGB, vigabatrin; TPM, topiramate; VPA, valproate; LTG, lamotrigine.

## Supplementary Materials

The following are available online at https://www.mdpi.com/2072-6643/12/8/2359/s1. Figure S1: Correlations between serum vitamin levels in refractory epilepsy patients; Table S1: Seizure severity and serum vitamin level of the patients at baseline and after 1, 3 and 6 months of multivitamin supplementation.

## References

[1] Kwan P., Brodie M.J. (2006). Refractory epilepsy: Mechanisms and solutions. Expert Rev. Neurother..

[2] Perucca P., Carter J., Vahle V., Gilliam F.G. (2009). Adverse antiepileptic drug effects: Toward a clinically and neurobiologically relevant taxonomy. Neurology.

[3] Kook D.H., Cho S.Y., Lee K.Y., Lee I.G., Lee J.S. (2006). A case of acute isoniazid intoxication in childhood. J. Korean Child Neurol. Soc..

[4] Dave H.N., Ramsay R.E., Khan F., Sabharwal V., Irland M. (2015). Pyridoxine deficiency in adult patients with status epilepticus. Epilepsy Behav..

[5] Ali A., Pillai K., Pal S.N. (2003). Effects of folic acid and lamotrigine therapy in some rodent models of epilepsy and behaviour. J. Pharm. Pharmacol..

[6] Rasic-Markovic A., Hrncic D., Krstic D., Colovic M., Djuric E., Rankov-Petrovic B., Susic V., Stanojlovic O., Djuric D. (2016). The effect of subchronic supplementation with folic acid and l-arginine on homocysteine-induced seizures. Can. J. Psychophysiol. Pharmacol..

[7] Holló A., Clemens Z., Kamondi A., Lakatos P., Szűcs A. (2012). Correction of vitamin D deficiency improves seizure control in epilepsy: A pilot study. Epilepsy Behav..

[8] Cannell J.J., Grant W.B. (2013). What is the role of vitamin D in autism?. Derm. Endocrinol..

[9] Borowicz K.K., Morawska D., Morawska M. (2015). Effect of cholecalciferol on the anticonvulsant action of some second generation antiepileptic drugs in the mouse model of maximal electroshock. Pharmacol. Rep..

[10] Ogunmekan A.O., Hwang P.A. (1989). A randomized, double-blind, placebo-controlled, clinical trial of D-alpha-tocopheryl acetate (vitamin E), as add-on therapy, for epilepsy in children. Epilepsia.

[11] Mehvari J., Motlagh F.G., Najafi M., Ghazvini M.R.A., Naeini A.A., Zare M. (2016). Effects of Vitamin E on seizure frequency, electroencephalogram findings, and oxidative stress status of refractory epileptic patients. Adv. Biomed. Res..

[12] Baluchnejadmojarad T., Roghani M. (2013). Coenzyme q10 ameliorates neurodegeneration, mossy fiber sprouting, and oxidative stress in intrahippocampal kainate model of temporal lobe epilepsy in rat. J. Mol. Neurosci..

[13] Sattarinezhad E., Shafaroodi H., Sheikhnouri K., Mousavi Z., Moezi L. (2014). The effects of coenzyme Q10 on seizures in mice: The involvement of nitric oxide. Epilepsy Behav..

[14] Tawfik M.K. (2011). Coenzyme Q10 enhances the anticonvulsant effect of phenytoin in pilocarpine-induced seizures in rats and ameliorates phenytoin-induced cognitive impairment and oxidative stress. Epilepsy Behav..

[15] Kwan P., Arzimanoglou A., Berg A.T., Brodie M.J., Allen Hauser W., Mathern G., Moshé S.L., Perucca E., Wiebe S., French J. (2010). Definition of drug resistant epilepsy: Consensus proposal by the ad hoc Task Force of the ILAE Commission on Therapeutic Strategies. Epilepsia.

[16] Gutch M., Kumar S., Razi S., Gupta K., Gupta A. (2015). Assessment of insulin sensitivity/resistance. Indian J. Endocrinol. Metab..

[17] Menik L., Palangasinghe S. Comparison of Insulin Resistance by Indirect Methods-HOMA, QUICKI and McAuley-with Fasting Insulin in Patients with Type 2 Diabetes in Galle, Sri Lanka: A Pilot Study. http://cogprints.org/5000/1/2006-1-2.pdf.

[18] Morisky D.E., Green L.W., Levine D.M. (1986). Concurrent and predictive validity of a self-reported measure of medication adherence. Med. Care.

[19] Lin Y.-S., Ho Y., Hu C.-J., Su W.-W., Hsu K.-Y., Shen W.W., Chiueh C.C., Yuan R.-Y. (2013). Development of a Taiwan version of the eight-item Morisky Medication Adherence Scale and factors influencing patients’ comprehension. J. Exp. Clin. Med..

[20] Tang F., Hartz A., Bauer B. (2017). Drug-resistant epilepsy: Multiple hypotheses, few answers. Front. Neurol..

[21] Thijs R.D., Surges R., O’Brien T.J., Sander J.W. (2019). Epilepsy in adults. Lancet.

[22] LoVecchio F., Curry S.C., Graeme K.A., Wallace K.L., Suchard J. (2001). Intravenous pyridoxine-induced metabolic acidosis. Ann. Emerg. Med..

[23] Wang X.L., Rainwater D.L., Mahaney M.C., Stocker R. (2004). Cosupplementation with vitamin E and coenzyme Q10 reduces circulating markers of inflammation in baboons. Am. J. Clin. Nutr..

[24] Huang C.W., Huang C.C., Wu S.N. (2006). The Opening Effect of Pregabalin on ATP-Sensitive Potassium Channels in Differentiated Hippocampal Neuron–derived H19-7 Cells. Epilepsia.

[25] Huang C.W., Huang C.C., Cheng J.T., Tsai J.J., Wu S.N. (2007). Glucose and hippocampal neuronal excitability: Role of ATP-sensitive potassium channels. J. Neurosci. Res..

[26] Huang C.-W., Tsai J.-J., Ou H.-Y., Wang S.-T., Cheng J.-T., Wu S.-N., Huang C.-C. (2008). Diabetic hyperglycemia is associated with the severity of epileptic seizures in adults. Epilepsy Res..

[27] Huang C.-W., Cheng J.-T., Tsai J.-J., Wu S.-N., Huang C.-C. (2009). Diabetic hyperglycemia aggravates seizures and status epilepticus-induced hippocampal damage. Neurotox. Res..

[28] Huang C.-W., Wu S.-N., Cheng J.-T., Tsai J.-J., Huang C.-C. (2010). Diazoxide reduces status epilepticus neuron damage in diabetes. Neurotox. Res..

[29] Effoe V.S., Wagenknecht L.E., Echouffo Tcheugui J.B., Chen H., Joseph J.J., Kalyani R.R., Bell R.A., Wu W.C.H., Casanova R., Bertoni A.G. (2017). Sex differences in the association between insulin resistance and incident coronary heart disease and stroke among blacks without diabetes mellitus: The Jackson Heart Study. J. Am. Heart Assoc..

[30] Alissa E.M. (2018). Relationship between serum gamma-glutamyltransferase activity and cardiometabolic risk factors in metabolic syndrome. J. Fam. Med Prim. Care.

[31] Panayiotou A., Kouis P., Griffin M., Nicolaides A. (2015). Comparison between insulin resistance indices and carotid and femoral atherosclerosis: A cross-sectional population study. Int. Angiol. J. Int. Union Angiol..

[32] Li J., Goh C.E., Demmer R.T., Whitcomb B.W., Du P., Liu Z. (2017). Association between serum folate and insulin resistance among US nondiabetic adults. Sci. Rep..

[33] Setola E., Monti L.D., Galluccio E., Palloshi A., Fragasso G., Paroni R., Magni F., Sandoli E.P., Lucotti P., Costa S. (2004). Insulin resistance and endothelial function are improved after folate and vitamin B12 therapy in patients with metabolic syndrome: Relationship between homocysteine levels and hyperinsulinemia. Eur. J Endocrinol..

[34] Szymczak-Pajor I., Śliwińska A. (2019). Analysis of Association between Vitamin D Deficiency and Insulin Resistance. Nutrients.

[35] Hoseini S.A., Ashraf Aminorroaya B.I., Amini M. (2013). The effects of oral vitamin D on insulin resistance in pre-diabetic patients. J. Res. Med. Sci. Off. J. Isfahan Univ. Med. Sci..

[36] Yu L., Zhai Y., Shen S. (2020). Association between vitamin D and prediabetes: A PRISMA-compliant meta-analysis. Medicine.

[37] Pott-Junior H., Nascimento C.M.C., Costa-Guarisco L.P., Gomes G.A.d.O., Gramani-Say K., Orlandi F.d.S., Gratão A.C.M., Orlandi A.A.d.S., Pavarini S.C.I., Vasilceac F.A. (2020). Vitamin D Deficient Older Adults Are More Prone to Have Metabolic Syndrome, but Not to a Greater Number of Metabolic Syndrome Parameters. Nutrients.

[38] Manning P.J., Sutherland W.H.F., Walker R.J., Williams S.M., de Jong S.A., Ryalls A.R., Berry E.A. (2004). Effect of High-Dose Vitamin E on Insulin Resistance and Associated Parameters in Overweight Subjects. Diabetes Care.

[39] Sanchez-Lugo L., Mayer-Davis E.J., Howard G., Selby J.V., Ayad M.F., Rewers M., Haffner S. (1997). Insulin sensitivity and intake of vitamins E and C in African American, Hispanic, and non-Hispanic white men and women: The Insulin Resistance and Atherosclerosis Study (IRAS). Am. J. Clin. Nutr..

[40] Schwetz V., Scharnagl H., Trummer C., Stojakovic T., Pandis M., Gruebler M.R., Verheyen N., Gaksch M., Zittermann A., Aberer F. (2018). Vitamin D supplementation and lipoprotein metabolism: A randomized controlled trial. J. Clin. Lipidol..

[41] Hodis H.N., Mack W.J., LaBree L., Mahrer P.R., Sevanian A., Liu C.-r., Liu C.-h., Hwang J., Selzer R.H., Azen S.P. (2002). Alpha-tocopherol supplementation in healthy individuals reduces low-density lipoprotein oxidation but not atherosclerosis: The Vitamin E Atherosclerosis Prevention Study (VEAPS). Circulation.

[42] Asbaghi O., Choghakhori R., Abbasnezhad A. (2019). Effect of Omega-3 and vitamin E co-supplementation on serum lipids concentrations in overweight patients with metabolic disorders: A systematic review and meta-analysis of randomized controlled trials. Diabetes Metab. Syndr. Clin. Res. Rev..

[43] Zhang P., Yang C., Guo H., Wang J., Lin S., Li H., Yang Y., Ling W. (2018). Treatment of coenzyme Q10 for 24 weeks improves lipid and glycemic profile in dyslipidemic individuals. J. Clin. Lipidol..

