# Supplementary material for: An Open Pilot Study of the Effect and Tolerability of Add-On Multivitamin Therapy in Patients with Intractable Focal Epilepsy
Source: Nutrients. 2020 Aug 7;12(8):2359. doi: 10.3390/nu12082359 (PMC7468939; doi:10.3390/nu12082359)

Table S1: Seizure severity and serum vitamin level of the patients at baseline and after 1, 3 and 6 months of multivitamin supplementation.

	Baseline	1 month	3 months	6 months
**Characteristics**	**Mean±SD**	**Mean±SD**	**Mean±SD**	**Mean±SD**
**Frequency, 28 days**	8.12±17.24	6.26±13.90	3.21±6.94	2.06±3.89
**Response, %**	0	-19.29±70.53	-39.53±55.99	-40.37±57.57
**Vitamins**				
**B6, nmol/L**	240.73±233.02	189.27±143.49	169.68±143.80	200.75±237.34
**B9, ng/ml**	8.26±4.13	37.88±22.63	56.62±52.84	48.40±32.38
**D, ng/ml**	27.70±13.91	30.92±12.52	34.17±11.89	33.90±11.55
**E, nmol/mL**	29.46±45.13	35.00±56.61	40.18±61.03	29.47±51.44
**Q10, ng/ml**	2,953.44±1,161.09	2,742.68±960.48	2,332.63±595.32	1,725.32±450.91

Data are presented as mean±SD. Abbreviations: B6, pyridoxal 5′-phosphate (PLP); B9, total folate; D, total 25-hydroxyvitamin D; E, total human vitamin E; Q10, total coenzyme Q10.

Figure S1: Correlations between serum vitamin levels in the refractory epilepsy patients.

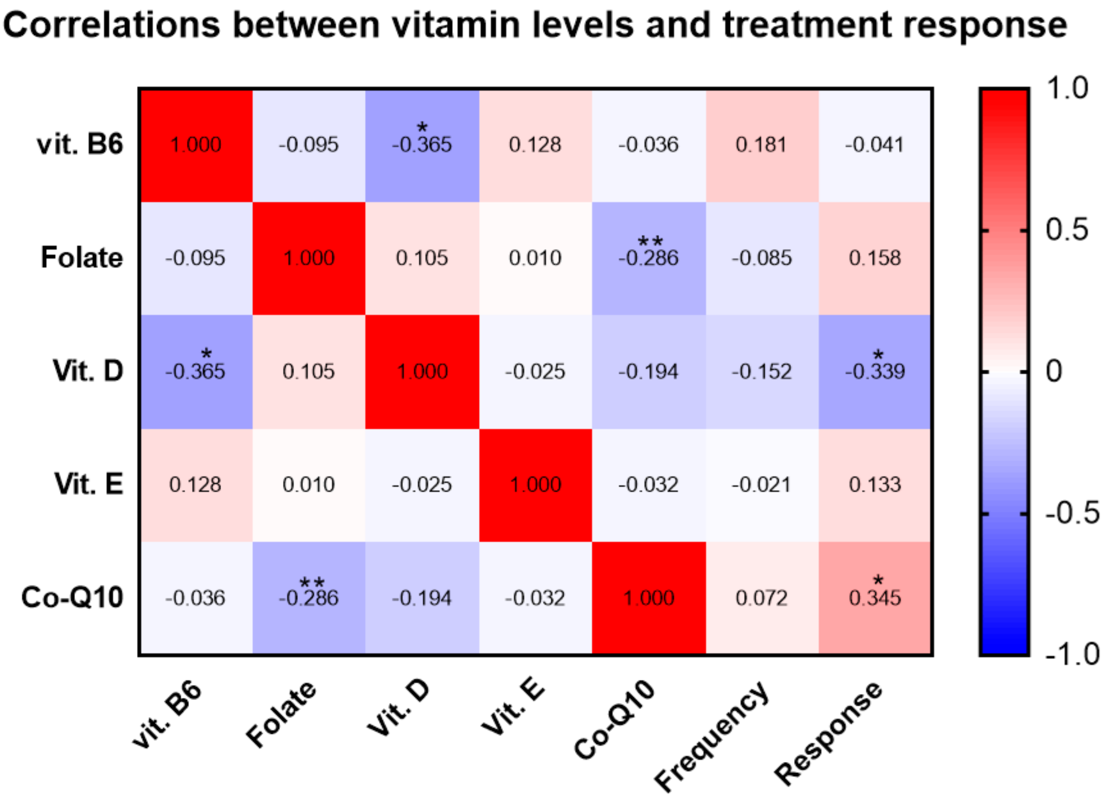

Supplement: Supplementary file 1 [file nutrients-12-02359-s001.pdf]
